# Peripheral perfusion, measured by perfusion index, is a novel indicator for renal events in patients with type 2 diabetes mellitus

**DOI:** 10.1038/s41598-020-62926-8

**Published:** 2020-04-08

**Authors:** Hiroshi Okada, Muhei Tanaka, Takashi Yasuda, Yuki Okada, Hisahiro Norikae, Tetsuya Fujita, Takashi Nishi, Hirokazu Oyamada, Tetsuro Yamane, Michiaki Fukui

**Affiliations:** 1 Department of Diabetes and Endocrinology, Matsushita Memorial Hospital, 5-55 Sotojima-cho, Moriguchi, 570-8540 Japan; 2 Department of Endocrinology and Metabolism, Kyoto Prefectural University of Medicine, Graduate School of Medical Science, Kyoto, Japan; 3 Department of Nephrology, Matsushita Memorial Hospital, Moriguchi, Japan; 4 Department of General Affairs, Matsushita Memorial Hospital, Moriguchi, Japan; 5 Department of Gastroenterology, Matsushita Memorial Hospital, Moriguchi, Japan; 6 Department of Surgery, Matsushita Memorial Hospital, Moriguchi, Japan

**Keywords:** Endocrinology, Nephrology

## Abstract

Diabetic kidney disease (DKD) is one of the leading causes of end stage renal disease. Despite recent therapies, mortality due to DKD and resources spent on healthcare are important problems. Thus, appropriate markers are needed to predict renal outcomes. Therefore, we investigated the role of peripheral perfusion as an indicator for renal events in patients with type 2 diabetes mellitus. This retrospective cohort study included 566 patients who were admitted to Matsushita Memorial Hospital in Osaka, Japan for type 2 diabetes mellitus. Peripheral perfusion was assessed using perfusion index (PI), which represents the level of circulation through peripheral tissues and was measured on each toe using a Masimo SET Radical-7 (Masimo Corporation, Irvine, CA, USA) instrument. The duration of follow up was 3.0 years. The median age of patients was 70 years (IQR range: 61–77 years) and median PI value was 2.9% (IQR range: 1.8–4.8%). Multiple logistic regression analyses showed that PI (per 1% increase) was associated with an odds ratio of composite of end-stage renal disease (ESRD) and/or doubling of serum creatinine level; n = 40 (odds ratio 0.823 [95% CI: 0.680–0.970]), and composite of ESRD, doubling of serum creatinine level, and renal death and/or cardiovascular death; n = 44 (odds ratio 0.803 [95% CI: 0.665–0.944]). The factors which were statistically significant in univariate analysis and those known to be related factors for renal event were considered simultaneously as independent variables for multiple logistic regression analysis. PI can be a novel indicator for renal events in patients with type 2 diabetes mellitus.

## Introduction

Despite the advances in renoprotective therapies, such as renin-angiotensin system blockade and sodium-glucose cotransporter-2 inhibitors^1,2^, the prevalence of diabetic kidney disease (DKD) and resources spent on healthcare due to DKD are increasing globally in patients with type 2 diabetes mellitus. DKD develops in approximately 40% of patients with diabetes and is one of the leading causes of end-stage renal disease^3–5^. DKD has been reported to be associated with the progression of cardiovascular diseases and increased risk of cardiovascular mortality^6–8^. Total deaths due to cardiovascular diseases and infections are highly prevalent in patients with end-stage renal disease (ESRD)^9^.

The progression of DKD involves glomerular hyperfiltration, albuminuria, decline in estimated glomerular filtration rate (eGFR), and ESRD. Patients with type 2 diabetes mellitus present heterogeneous process of DKD^10^. Indeed, normoalbuminuric renal insufficiency is common in patients with type 2 diabetes mellitus^11^. Interstitial changes such as chronic renal hypoxia might precede glomerular disease without albuminuria, wherein, intrarenal arteriosclerosis might be a main factor for declining eGFR. Further, the peripheral perfusion index (PI) is the ratio of pulsatile blood flow to non-pulsatile blood flow in the monitored tissue and has been shown to reflect changes in peripheral perfusion^12,13^. We have recently demonstrated that a low PI value indicates atherosclerosis in clinical care setting^14^ and diabetic kidney disease in the patients with type 2 disease in the cross sectional study^15^. However, the association of diminished PI with renal events in patients with type 2 diabetes mellitus has not been reported. Therefore, we aimed to clarify whether PI might be an indicator for renal events including decline in eGFR, cardiovascular death, and progression of albuminuria, in patients with type 2 diabetes mellitus.

## Results

The characteristics of 566 patients enrolled in this study are shown in Table 1. The median age of patients was 70 years (IQR range: 61–77 years) and the median PI value was 2.9% (IQR range: 1.8–4.8%). The number of patients who developed ESRD, showed doubling of serum creatinine level, renal death and cardiovascular death, and the progression of albuminuria during the study period was 14 (2.5%), 38 (6.7%), 10 (1.8%), and 192 (33.9%), respectively. The number of patients who developed composite of ESRD, and/or doubling of serum creatinine level and composite of ESRD, doubling of serum creatinine level, renal death and/or cardiovascular death was 40 (7.1%) and 44 (7.8%), respectively.

**Table 1:** Characteristics of patients.

	All	Patients with eGFR ≥ 60 ml/min/1.73 m^2^
n (male/female)	566 (332/234)	358 (214/144)
Age (years)	70 (61–77)	67 (54–75)
Duration of diabetes (years)	7.0 (4.0-14.0)	6.0 (4.0–13.0)
Body mass index (kg/m^2^)	24.3 (21.54–27.8)	23.9 (20.9–27.3)
Systolic blood pressure (mmHg)	129 (115–143)	129 (117–141)
Diastolic blood pressure (mmHg)	75 (65–82)	76 (68–82)
Heart rate (beat/min)	78 (70–90)	79 (71–88)
Perfusion index (%)	2.9 (1.8–4.8)	3.2 (2.1–5)
Hemoglobin A1c (%)	8.4 (7.3–9.9)	8.5 (7.4–10.0)
Total cholesterol (mg/dl)	177 (155–209)	177 (157–210)
Triglycerides (mg/dl)	126 (87.8–189.3)	123 (81–181)
Uric acid (mg/dl)	5.0 (4.0–6.2)	4.5 (3.7–5.5)
Creatinine (mg/dl)	0.8 (0.65–1.03)	0.69 (0.56–0.81)
eGFR (ml/min/1.73 m^2^)	66.2 (51.1–81.3)	78.6 (67.8–93.7)
UACR (mg/g Cr)	37.2 (9.5–138.5)	21.7 (8.7–58.2)
Hypertension (−/+)	254/312	188/170
Smoking status (never/past/recent)	252/116/198	146/78/134
History of cardiovascular disease (−/+)	378/188	269/89
RAS inhibitor (−/+)	316/250	224/134
Incretin related therapies (−/+)	170/396	128/230
SGLT-2 inhibitor (−/+)	522/44	326/32
Statin (−/+)	336/230	232/126

Data are expressed as the median (interquartile range) or absolute number.

eGFR, estimated glomerular filtration rate; UACR, urine albumin-to-creatinine ratio; RAS, renin-angiotensin system; SGLT, sodium-glucose cotransporter

Table 2 shows correlation between PI values and other variables. BMI, total cholesterol, and logarithm of triglyceride level were positively correlated with PI. Age, creatinine level, and logarithm of UACR were negatively correlated with PI. Tables 3 and 4 show unadjusted and adjusted odds ratios of PI for composite of ESRD and/or doubling of serum creatinine level (Tables 3a and 4a), composite of end-stage renal disease, doubling of serum creatinine level, renal death and/or cardiovascular death (Tables 3b and 4b) and progression of albuminuria (Tables 3c and 4c). Table 3 includes all patients, while Table 4 includes the patients with eGFR ≥ 60 ml/min/1.73 m^2^. Multiple logistic regression analyses showed that PI (per 1% increase) was associated with an odds ratio of composite of ESRD and/or doubling of serum creatinine level in all patients as well as in patients with eGFR ≥ 60 ml/min/1.73 m^2^ (odds ratio 0.823 [95% CI: 0.680–0.970] or 0.598 [95% CI: 0.363–0.871]), and composite of ESRD, doubling of serum creatinine level, and renal death and/or cardiovascular death (odds ratio 0.803 [95% CI: 0.665–0.944] or 0.662 [95% CI: 0.458–0.891]). PI value was associated with an odds ratio of progression of albuminuria only in crude model. Moreover, multiple logistic regression analyses (Model 3) in all patients showed that baseline serum creatinine level was associated with an odds ratio of composite of ESRD and/or doubling of serum creatinine level (odds ratio 2.228 [95% CI: 0.987–5.023]), logarithm of baseline UACR was associated with an odds ratio of composite of ESRD, doubling of serum creatinine level, and renal death and/or cardiovascular death (odds ratio 1.678 [95% CI: 1.074–2.642]), and duration of diabetes, logarithm of baseline UACR or history of cardiovascular disease was associated with an odds ratio of progression of albuminuria (odds ratio 1.054 [95% CI: 1.021–1.089], odds ratio 1.452 [95% CI: 1.115–1.897] or odds ratio 1.845 [95% CI: 1.170–2.940]), respectively. Multiple logistic regression analyses (Model 3) in patients with eGFR ≥ 60 ml/min/1.73 m^2^ showed that BMI was associated with an odds ratio of composite of ESRD and/or doubling of serum creatinine level (odds ratio 1.169 [95% CI: 1.033–1.330]) and duration of diabetes, baseline serum creatinine level (per 0.1 mg/dl increase), logarithm of baseline UACR or history of cardiovascular disease was associated with an odds ratio of progression of albuminuria (odds ratio 1.079 [95% CI: 1.031–1.130], odds ratio 1.326 [95% CI: 1.075–1.647], odds ratio 1.655 [95% CI: 1.141–2.415] or odds ratio 2.452 [95% CI: 1.233–5.021]), respectively.

**Table 2:** Correlation between perfusion index and other variables.

	β	*P*
Age	−0.210	<0.0001
Duration of diabetes	−0.081	0.054
Body mass index	0.157	0.0002
Systolic blood pressure	0.004	0.934
Diastolic blood pressure	0.078	0.065
Heart rate	−0.063	0.137
Hemoglobin A1c	0.033	0.211
Total cholesterol	0.123	0.004
Logarithm of triglycerides	0.109	0.011
Uric acid	0.008	0.853
Creatinine	−0.145	0.0005
Logarithm of urine albumin-to-creatinine ratio	−0.124	0.003

**Table 3:** Model 1: age, duration of diabetes, body mass index, systolic blood pressure, hemoglobin A1c, total cholesterol, uric acid. Model 2: Model 1 plus creatinine. Model 3: Model 2 plus sex, logarithm of urine albumin-to-creatinine ratio, current or past smoker, history of cardiovascular disease, renin-angiotensin system inhibitor, incretin related therapies, sodium-glucose cotransporter-2 inhibitor, statin.

	Odds ratio	95% CI	P
**a. Unadjusted and adjusted odds ratios of perfusion index for composite of end-stage renal disease and/or doubling of serum creatinine level in all patients**.			
Crude	0.830	0.698–0.963	0.023
Model 1	0.821	0.684–0.959	0.011
Model 2	0.847	0.708–0.989	0.035
Model 3	0.823	0.680–0.970	0.018
**b. Unadjusted and adjusted odds ratios of perfusion index for composite of end-stage renal disease, doubling of serum creatinine level, renal death and/or cardiovascular death in all patients**			
Crude	0.812	0.685–0.940	0.004
Model 1	0.801	0.671–0.934	0.003
Model 2	0.822	0.689–0.958	0.010
Model 3	0.803	0.665–0.944	0.007
**c. Unadjusted and adjusted odds ratios of perfusion index for progression of albuminuria in all patients**			
Crude	0.918	0.854–0.983	0.017
Model 1	0.943	0.874–1.014	0.119
Model 2	0.946	0.877–1.018	0.142
Model 3	0.945	0.872–1.021	0.154

**Table 4:** .

	Odds ratio	95% CI	*P*
**a. Unadjusted and adjusted odds ratios of perfusion index for composite of end-stage renal disease and/or doubling of serum creatinine level in patients with eGFR ≥ 60 ml/min/1.73 m**^**2**^.			
Crude	0.670	0.485–0.870	0.007
Model 1	0.638	0.430–0.864	0.002
Model 2	0.641	0.428–0.876	0.003
Model 3	0.598	0.363–0.871	0.004
**b. Unadjusted and adjusted odds ratios of perfusion index for composite of end-stage renal disease, doubling of serum creatinine level, renal death and/or cardiovascular death in patients with eGFR ≥ 60 ml/min/1.73 m**^**2**^			
Crude	0.688	0.511–0.877	0.007
Model 1	0.684	0.490–0.891	0.003
Model 2	0.687	0.488–0.899	0.004
Model 3	0.662	0.458–0.891	0.004
**c. Unadjusted and adjusted odds ratios of perfusion index for progression of albuminuria in patients with eGFR ≥ 60 ml/min/1.73 m**^**2**^			
Crude	0.907	0.829–0.986	0.027
Model 1	0.959	0.869–1.049	0.352
Model 2	0.954	0.867–1.047	0.324
Model 3	0.926	0.830–1.027	0.147

Model 1: age, duration of diabetes, body mass index, systolic blood pressure, hemoglobin A1c, total cholesterol, uric acid.

Model 2: Model 1 plus creatinine.

Model 3: Model 2 plus sex, logarithm of urine albumin-to-creatinine ratio, current or past smoker, history of cardiovascular disease, renin-angiotensin system inhibitor, incretin related therapies, sodium-glucose cotransporter-2 inhibitor, statin.

Net reclassification index (NRI) and integrated discrimination improvement (IDI) was derived from logistic regression models (Model 3) with and without logarithm of baseline UACR. Both NRI and IDI suggested that including baseline UACR in Model 3 results in significant improvement in performance. The NRI or IDI was estimated at 0.484 (95% CI: 0.176–0.792), 0.567 (95% CI: 0.210–0.803) and 0.167 (95% CI: −0.012–0.345) or 0.010 (95% CI: 0.0008–0.019), 0.017 (95% CI: 0.004–0.030) and 0.013 (95% CI: 0.003–0.022) in analyses for composite of ESRD and/or doubling of serum creatinine level, composite of end-stage renal disease, doubling of serum creatinine level, renal death and/or cardiovascular death and progression of albuminuria in all patients, respectively.

## Discussion

The major finding of our study is that PI, which represents peripheral perfusion, can be a novel indicator for renal events such as composite of ESRD, doubling of serum creatinine level, renal death and/or cardiovascular death in patients with type 2 diabetes mellitus.

The United Kingdom Prospective Diabetes Study showed that 40% patients developed albuminuria, while 30% patients had eGFR < 60 ml/min/1.73 m^2^ or doubling of the blood creatinine level at a median duration of 15 years after diagnosis^9,15^. Patients with ESRD had high prevalence of mortality due to cardiovascular diseases and infections. The overall death rate in patients with ESRD, having creatinine levels >2 mg/dL or those receiving kidney replacement therapy was nearly 20% per year^9^. In spite of the development of recent therapies, large residual risks of renal events remain. Therefore, an easy and useful tool is required to improve renal outcomes in patients with type 2 diabetes mellitus. The present study suggests that PI could be a useful tool to indicate renal events in patients with type 2 diabetes mellitus.

In our study, PI was strongly associated with decline in eGFR, composite of ESRD and/or doubling of serum creatinine level. However, multiple regression analysis suggested that PI was not associated with progression of albuminuria. Recently, we have reported the association PI and diabetic kidney disease in the cross sectional study^15^. Low PI value was more associated with eGFR < 60 mL/min per 1.73 m^2^ than albuminuria, which is consistent with our results. This can be explained by the mechanism and structural changes during decline of eGFR and progression of albuminuria. The initial structural change during DKD is thickening of glomerular basement membrane along with capillary and tubular basement membrane thickening^16–18^. Further, glomerular changes include loss of endothelial fenestrations, mesangial matrix expansion, and loss of podocytes^16,17^. It has been reported that albuminuria is correlated with mesangial expansion^3^, which is a marker for glomerular disease and an early clinical indicator of DKD. Diabetic control has been considered as an important factor for prevention of albuminuria^16^. Previous studies have shown high prevalence of patients with type 2 diabetes mellitus having reduced eGFR without albuminuria. Yokoyama *et al*.^19^ reported that proportion of patients with normoalbuminuria was 52% among patients with eGFR < 60 ml/min/1.73 m^2^. Chronic hypoxia in tubulointerstitium can explain the decline in eGFR without the presence of albuminuria. Chronic hypoxia in tubulointerstitium results in advanced tubulointerstitial lesions besides minor diabetic glomerular lesions^20,21^. Some mechanisms can explain development of chronic hypoxia in tubulointerstitium. One of the mechanisms is diminished microcirculation, such as distortion and loss of peritubular capillaries due to intrarenal arteriosclerosis^22–26^. These changes may induce a vicious cycle of regional hypoxia and decline in eGFR. Taken together, decrease in eGFR in patients without the presence of albuminuria is mainly associated with atherosclerosis. We have recently reported that low value of PI was strongly correlated with atherosclerosis^14^. Low value of PI can denote low peripheral perfusion resulting from atherosclerosis. Therefore, PI can be associated with decline in eGFR and not with progression of albuminuria. We have also reported that PI was associated with ankle brachial index^14^. Lower ankle brachial index has been associated with cardiovascular mortality^27^. Therefore, PI may be strongly associated with composite renal event including cardiovascular death.

In our study, PI is directly correlated with total cholesterol and triglycerides. Moreover, PI is negatively correlated with age and positively correlated with BMI and serum albumin (r = 0.154, *p* = 0.0003). We postulated that PI was also affected by nutrition status. We supposed that PI could be affected by atherosclerosis. However, PI is not correlated with systolic blood pressure, which is consisted with recent report^14^. This could be explained by the effect of some factors such as duration of hypertension or the effect of antihypertensive medications.

This study has several limitations. It is uncertain if the results of our study are applicable to people of other ethnicities because we only targeted Japanese patients. Moreover, because the number of patients was limited, the volume of information gathered may be insufficient. Patients were old, with small duration of diabetes, mainly hypertensives and poorly controlled in our study. Moreover, change of treatment for hypertension or for diabetes or any other treatment could affect atherosclerosis. Unfortunately, however, we have insufficient data about change of treatment for hypertension or for diabetes or any other treatment during study period. Although our investigation shows that PI may be a useful indicator of renal events, further investigation using a larger population with patients from various ethnicities would be crucial to predict renal events.

PI might have an important role in the prevention of renal events; however, interventional methods to improve PI have not been established in humans. Patients with low value of PI may require aggressive lifestyle modifications and medication to lower blood glucose and blood pressure for the prevention of renal events. To the best of our knowledge, this is the first study to investigate if PI could be an indicator for renal events in patients with type 2 diabetes mellitus. In conclusion, PI may be a valuable indicator of renal events in patients with type 2 diabetes mellitus.

## Materials and Methods

### Ethics

This study was approved by local research ethics committee of Matsushita Memorial Hospital and has been conducted in accordance with the ethical principles of the Declaration of Helsinki. Written informed consent was obtained from all of the patients.

### Patients and data collection

This was a retrospective cohort study conducted in 566 patients admitted to Matsushita Memorial Hospital in Osaka, Japan for treatment of type 2 diabetes mellitus between September 2015 and October 2016. The duration of follow up was 3.0 years. All data were retrieved from the database. Fasting blood samples were collected in the morning. Total serum cholesterol and triglyceride concentrations were assessed using standard enzymatic methods.

Diabetes was diagnosed based on the report of the expert committee on the diagnosis and classification of diabetes mellitus^28,29^. Hypertension was defined when the patient’s systolic and diastolic blood pressure was greater than 140 mm Hg and 90 mm Hg, respectively, and/or the patient was prescribed any antihypertensive medications. Patients were classified as non-smokers, past smokers, or current smokers based on the self-administered questionnaire. eGFR was calculated using the Japanese Society of Nephrology equation(30): eGFR = 194 × Cre^−1.094^ × age^−0.287^ (ml/min/1.73 m^2^). For women, the value of eGFR was multiplied by a correction factor of 0.739. ESRD was defined by the initiation of dialysis or eGFR < 15 ml/min/1.73 m^2^. The history of cardiovascular disease was defined as the patients with the history of myocardial infarction, heart failure, or stroke. Renal death was defined as death with a proximate renal cause. Death due to myocardial infarction, heart failure, or stroke was defined as cardiovascular death. Urine albumin-to-creatinine ratio (UACR) was determined using early morning urine. UACR was measured using an immunoturbidimetric assay. Microalbuminuria was defined by UACR 30 to < 300 mg/g Cr, whereas, macroalbuminuria was defined by UACR ≥ 300 mg/g Cr. Progression of albuminuria was defined as >30% increase in albuminuria, and a change from normoalbuminuria to either microalbuminuria or macroalbuminuria, or from microalbuminuria to macroalbuminuria. Patients for whom PI measurement could not be obtained were excluded from this study. Other exclusion criteria included patients with implanted cardiac pacemakers; arrhythmia, such as paroxysmal atrial fibrillation; amputations in any part of the lower extremities; and patients with malignancy or ESRD at baseline.

### Technique for determining PI

PI was measured using a Masimo SET Radical-7 (Masimo Corporation, Irvine, CA, USA) instrument. The patients were placed in the supine position. A Masimo pulse oximeter probe was positioned on each toe and connected to Masimo SET Radical-7 machine. The patients were asked to rest for 5 min at the beginning of the procedure. PI was recorded three times: at 20 s, 40 s, and 60 s, after the 5-min rest period. The average of the three values was calculated and used as the reference value. The Masimo SET Radical-7 calculates PI as the ratio between the pulsatile and non-pulsatile components of the light reaching a light-sensitive cell of the pulse oximetry probe. The reliability and reproducibility of PI have been reported elsewhere^14,30^. After bilateral determination, the lower value of PI was considered as a representative for each subject.

### Statistical analysis

The median and frequency of potential confounding variables were calculated. The relationships between PI and age, BMI, and other variables at baseline were estimated using Spearman’s rank correlation analyses. Owing to the relationship between baseline renal function and development of renal events, we also conducted analyses in a subgroup of patients with eGFR ≥ 60 ml/min/1.73 m^2^. Logistic regression analyses were conducted to examine the effects of PI on composite of ESRD, and/or doubling of serum creatinine level, composite of end-stage renal disease, doubling of serum creatinine level, renal death and/or cardiovascular death, and progression of albuminuria in all patients and in patients with eGFR ≥ 60 ml/min/1.73 m^2^. The following confounding factors were considered: age, duration of diabetes, body mass index, systolic blood pressure, hemoglobin A1c, total cholesterol and uric acid (Model 1). Model 2 was adjusted for all variables in Model 1 plus creatinine level. Model 3 was adjusted for all variables in Model 2 plus sex, logarithm of UACR, smoking status, history of cardiovascular disease, the use of renin-angiotensin system inhibitor, incretin related therapies, sodium-glucose cotransporter-2 inhibitor and statins. NRI and IDI were derived from logistic regression models (Model 3) with and without logarithm of baseline UACR to evaluate the clinical impact.

All continuous variables are presented as median (interquartile range) or absolute number. *P*-value <0.05 was considered as statistically significant. The size, direction, and statistical significance of relationships were estimated by the odds ratio with 95% confidence interval (CI).

## Data Availability

The datasets generated during the current study are available from the corresponding author on reasonable request.

## References

[1] Brenner BM (2001). RENAAL Study Investigators. Effects of losartan on renal and cardiovascular outcomes in patients with type 2 diabetes and nephropathy. N. Engl. J. Med..

[2] Perkovic V (2019). CREDENCE Trial Investigators. Canagliflozin and Renal Outcomes in Type 2 Diabetes and Nephropathy. N. Engl. J. Med..

[3] Alicic RZ, Rooney MT, Tuttle KR (2017). Diabetic Kidney Disease: Challenges, Progress, and Possibilities. Clin. J. Am. Soc. Nephrol..

[4] USRDS: United States Renal Data System Annual Data Report: Epidemiology of Kidney Disease in the United States, Bethesda, MD, National Institute of Diabetes and Digestive and Kidney Diseases, https://www.usrds.org/2016/view/Default.aspx (2016).

[5] Reutens AT (2013). Epidemiology of diabetic kidney disease. Med. Clin. North. Am..

[6] Go AS, Chertow GM, Fan D, McCulloch CE, Hsu CY (2004). Chronic kidney disease and the risks of death, cardiovascular events, and hospitalization. N. Engl. J. Med..

[7] Drury PL (2011). Estimated glomerular filtration rate and albuminuria are independent predictors of cardiovascular events and death in type 2 diabetes mellitus: the Fenofibrate Intervention and Event Lowering in Diabetes (FIELD) study. Diabetologia..

[8] Dinneen SF, Gerstein HC (1997). The association of microalbuminuria and mortality in non-insulin-dependent diabetes mellitus. A systematic overview of the literature. Arch. Intern. Med..

[9] Adler AI (2003). UKPDS GROUP: Development and progression of nephropathy in type 2 diabetes: The United Kingdom Prospective Diabetes Study (UKPDS 64). Kidney Int..

[10] Fioretto P, Caramori ML, Mauer M (2008). The kidney in diabetes: Dynamic pathways of injury and repair. The Camillo Golgi Lecture 2007. Diabetologia..

[11] Shimizu M (2014). Kidney lesions in diabetic patients with normoalbuminuric renal insufficiency. Clin. Exp. Nephrology..

[12] Lima AP, Beelen P, Bakker J (2002). Use of a peripheral perfusion index derived from the pulse oximetry signal as a noninvasive indicator of perfusion. Crit. Care Med..

[13] Galvin EM (2006). Peripheral flow index is a reliable and early indicator of regional block success. Anesth. Analg..

[14] Okada H (2019). The perfusion index is a useful screening tool for peripheral artery disease. Heart Vessels..

[15] Retnakaran R, Cull CA, Thorne KI, Adler AI, Holman RR (2006). UKPDS Study Group: Risk factors for renal dysfunction in type2 diabetes: U. K. Prospective Diabetes Study 74. Diabetes..

[16] Caramori ML, Parks A, Mauer M (2013). Renal lesionspredictprogression of diabetic nephropathy in type 1 diabetes. J. AmSoc Nephrol..

[17] Fioretto P, Mauer M (2007). Histopathology of diabetic nephropathy. Semin. Nephrol..

[18] Tyagi I, Agrawal U, Amitabh V, Jain AK, Saxena S (2008). Thickness of glomerular and tubular basement membranes in preclinical and clinical stages of diabetic nephropathy. Indian. J. Nephrol..

[19] Yokoyama H (2009). Japan Diabetes Clinical Data Management Study Group. Prevalence of albuminuria and renal insufficiency and associated clinical factors in type 2 diabetes: the Japan Diabetes Clinical Data Management study (JDDM15). Nephrol. Dial. Transplant..

[20] Taniwaki H (1998). Decrease in glomerular filtration rate in Japanese patients with type 2 diabetes is linked to atherosclerosis. Diabetes Care..

[21] MacIsaac RJ (2006). Is nonalbuminuric renal insufficiency in type 2 diabetes related to an increase in intrarenal vascular disease?. Diabetes Care..

[22] Choi YJ (2000). Peritubular capillary loss is associated with chronic tubulointerstitial injury in human kidney: Altered expression of vascular endothelial growth factor. Hum. Pathol..

[23] Ohashi R, Kitamura H, Yamanaka N (2000). Peritubular capillary injury during the progression of experimental glomerulonephritis in rats. J. Am. Soc. Nephrol..

[24] Kairaitis LK, Wang Y, Gassmann M, Tay YC, Harris DC (2005). HIF-1alpha expression follows microvascular loss in advanced murine adriamycin nephrosis. Am. J. Physiol. Ren. Physiol..

[25] Yuan H-T, Li X-Z, Pitera JE, Long DA, Woolf AS (2003). Peritubular capillary loss after mouse acute nephrotoxicity correlates with down-regulation of vascular endothelial growth factor-A and hypoxia-inducible factor-1alpha. Am. J. Pathol..

[26] Bohle A, von Gise H, Mackensen-Haen S, Stark-Jakob B (1981). The obliteration of the postglomerular capillaries and its influence upon the function of both glomeruli and tubuli. Funct. interpretation morphologic findings. Klin. Wochenschr..

[27] Diehm C (2009). German Epidemiological Trial on Ankle Brachial Index Study Group. Mortality and vascular morbidity in older adults with asymptomatic versus symptomatic peripheral artery disease. Circulation..

[28] Expert Committee on the Diagnosis and Classification of Diabetes Mellitus Report of the expert committee on the diagnosis and classification of diabetes mellitus. *Diabetes Care*. **26**, 5–20 (2003).

[29] Matsuo S (2009). Collaborators developing the Japanese equation for estimated GFR: Revised equations for estimated GFR from serum creatinine in Japan. Am. J. Kidney Dis..

[30] Quinn CT, Raisis AL, Musk GC (2013). Evaluation of Masimo signal extraction technology pulse oximetry in anaesthetized pregnant sheep. Vet. Anaesth. Analg..

